# Capturing RNA-dependent pathways for cryo-EM analysis

**DOI:** 10.5936/csbj.201204003

**Published:** 2012-02-23

**Authors:** Justin R. Tanner, Katherine Degen, Brian L. Gilmore, Deborah F. Kelly

**Affiliations:** a Virginia Tech Carilion Research Institute, Roanoke, VA, 24016, USA; b Department of Biomedical Engineering, University of Virginia, Charlottesville, VA, 22908, USA

**Keywords:** Protein synthesis, Transcription, Lipid monolayer, Affinity Capture technology

## Abstract

Cryo-Electron Microscopy (EM) is a powerful technique to visualize biological processes at nanometer resolution. Structural studies of macromolecular assemblies are typically performed on individual complexes that are biochemically isolated from their cellular context. Here we present a molecular imaging platform to capture and view multiple components of cellular pathways within a functionally relevant framework. We utilized the bacterial protein synthesis machinery as a model system to develop our approach. By using modified Affinity Grid surfaces, we were able to recruit multiple protein assemblies bound to nascent strands of mRNA. The combined use of Affinity Capture technology and single particle electron microscopy provide the basis for visualizing RNA-dependent pathways in a remarkable new way.

## Introduction

Bacterial protein synthesis occurs in a continuum where transcriptional and translational events are coupled by RNA messages. Joining the two processes is thought to prevent toxic or non-functional mRNAs from accumulating inside the cell [1, 2]. Cryo-Electron Microscopy (EM) has been used extensively to study these intricate mechanisms (i.e. [3–5]. While many EM studies are focused on determining high-resolution structures of individual complexes [6–8], little is known of how multiple players collectively form biological pathways. In fact, many biochemical experiments are performed on macromolecular complexes far removed from their cellular context. This results in static snapshots of dynamic processes, which are highly informative for 3-D structure determination. The heterogeneous nature of biological specimens, however, is particularly useful in interpreting subtle changes that can be delineated by single particle EM [9, 10]. Our presented work demonstrates a unique strategy for capturing and imaging multiple, heterogeneous protein complexes that constitute biological pathways. We utilize the machinery that drives bacterial protein production as a model system to examine the molecular landscape of functional RNA-dependent pathways.

**Figure 1:**
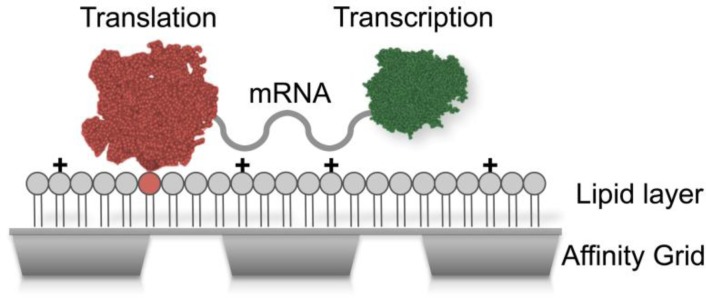
**Affinity Capture approach for recruiting multiple biological complexes**. Affinity Grids were coated with a monolayer containing functionalized Ni-NTA lipids (red) and positively charged (**+**) additives. Translational complexes (red) were specifically recruited to the grid while charged elements stabilize nascent mRNA coupled to transcriptional complexes (green).

The monolayer purification technique was recently developed in the EM field to study macromolecular complexes from cell lysates using single particle analysis [11]. This nanoscale purification system exploits lipid monolayers containing functionalized Nickel-nitrilotriacetic acid (Ni-NTA) lipids to concentrate proteins in low abundance. An extension of this technique gave rise to Affinity Grids, EM grids with a pre-deposited Ni-NTA lipid layer [12]. Our previous studies demonstrated the feasibility of imaging functional translational machinery actively bound to native mRNA. We now show that the incorporation of nucleotide stabilizing elements into our affinity matrix allows for the recruitment of multiple protein assemblies bound to the same mRNA transcript (Figure 1). This provides a new approach for studying protein-nucleotide interactions at nanometer resolution.

## Experimental Procedures

### Expression of His-tagged translational complexes in E. coli

A construct (clone MPMGp800 I10582) containing His-tagged ribosomal subunit 3, rpl3, was purchased from RZPD (GmbH, Berlin, Germany). The construct contained the T7 promoter in the PQE30NST vector. Rpl3 was expressed in *E. coli* (BL21 strain) overnight at 16°C in 100 ml of LB media containing 3 mg/l kanamycin and 50 mg/l ampicillin upon the addition of 0.5 mM IPTG at an OD_600_ = 0.6.

### Preparation of bacterial cell extract

The *E. coli* cells expressing rpl3 were harvested and centrifuged at 3,500g for 20 minutes at 4°C. Cell pellets from 100 ml of culture were suspended in 10 ml of lysis buffer containing 20 mM Hepes, pH 7.5, 150 mM NaCl and 0.2 g of lysozyme (EMD Biosciences, Inc., San Diego, CA) followed by sonication. The cell extract was centrifuged at 15,000g for 20 minutes at 4°C. The total protein concentration of the cleared supernatant was determined to be ∼ 4 mg/ml using a standard BCA protein assay (Pierce, Rockford, IL). The bacterial lysate was diluted to 0.2 mg/ml prior to use in buffer containing 20 mM Hepes, pH 7.5, 150 mM NaCl, 20 mM MgCl_2_, 20 mM CaCl_2_ and 60 mM imidazole.

### Preparation of Affinity Grid specimens

For negatively stained Affinity Grid specimens, 25-µl aliquots of Milli-Q water were placed in the wells of Teflon blocks and overlaid with a monolayer containing 1,2-Dilauroyl- phosphocholine (DLPC) (Avanti Polar Lipids, Alabaster, AL), 1,2-dioleoyl- iminodiacetic acid-succinyl-nickel salt (Ni-NTA lipid) (Avanti Polar Lipids, Alabaster, AL) and varying amounts of Didodecyldimethylammonium bromide (DDMA) (Sigma-Aldrich, St. Louis, MO). For cryo-EM specimens, 20% Ni-NTA lipids and 10% DDMA were used in the lipid monolayer mixture along with DLPC filler lipids. Lipid monolayers were incubated for 60 minutes on ice after which time EM grids were applied to the monolayer surface and lifted off following a 1-minute incubation. In the case of negatively stained specimens, continuous carbon grids were used. In the case of cryo specimens, Quantifoil (Quantifoil Micro Tools, GmbH, Germany) 200 mesh holey carbon grids with 2 µm holes and 2 µm spacing between holes were used. The excess solution was removed from each grid using a Hamilton syringe and 3-µl aliquots of the cleared bacterial lysate containing 60 mM imidazole was added to each Affinity Grid and incubated for 2 minutes. In our time course experiment, samples were incubated on the grids for 2, 5, 10 and 20 minutes.

For RNase A experiments, cell lysate samples in solution were incubated in the presence and absence of 10 units of RNase A (Roche Diagnostics, Indianapolis, IN) for 30 minutes at 4°C. Samples were then applied to Ni-NTA containing Affinity Grids in the presence and absence of DDMA and incubated for 2 minutes. Specimens were then prepared by negative staining using 1% uranyl formate [13] or by plunging into liquid ethane after blotting for 3 seconds using a Vitrobot (FEI Company, Hillsboro, OR).

### Western blot analysis

To detect the presence of RNA polymerase in our EM samples, specimens were prepared on 20% Ni-NTA Affinity Grids, with or without 10% DDMA. Bacterial lysates were incubated with the Affinity Grids for 2 minutes. Following this incubation, the excess solution was removed from each grid which was then incubated with 5 µl of elution buffer containing 20 mM Hepes, pH 7.5, 150 mM NaCl, 20 mM MgCl_2_, 20 mM CaCl_2_ and 200 mM imidazole. The 5-µl drop of eluate was recovered using a pipetteman and combined with additional eluates from a total of 10 grids for each condition tested. Recovered eluates were applied to a 10% SDS-PAGE gel. Following electrophoresis, western blot analysis was performed. RNA Polymerase β, the 146-kDa core protein subunit of bacterial RNA polymerase, was detected by western blot using anti-RNA Pol β (Santa Cruz Biotechnology, Inc., Santa Cruz, CA) and developed by the horseradish peroxidase method using the ECL Prime detection kit (GE Healthcare, Piscataway, NJ).

### Electron microscopy

Negatively stained specimens were imaged in an FEI Spirit BioTwin Electron Microscope (FEI, Hillsboro, OR) equipped with a tungsten filament and operated at an acceleration voltage of 120 kV under low-dose conditions. Images were recorded on a FEI Eagle 2k HS CCD camera with a pixel size of 30 µm at a nominal magnification of 68,000x and a defocus value of -1.5 µm for a sampling of 4.4 Å/pixel. Grids of frozen-hydrated specimens were transferred into the electron microscope using a Gatan 626 cryo-specimen holder, maintaining a temperature of -180°C. Samples were examined using low-dose procedures and images were recorded at a nominal magnification of 50,000x using a defocus value of -3 µm for a sampling of 6 Å/pixel.

### Image processing

Using the WEB interface associated with the SPIDER software package [14], 12,510 particles were selected from 200 images of vitrified specimens of bacterial lysate purified on Affinity Grids containing DDMA added to the lipid layer. Particles were windowed into individual images of 90 x 90 pixels. Using the SPIDER software package, each set of particles was subjected to 10 cycles of multi-reference alignment. The references used for the first multi-reference alignment were randomly selected from the raw images. Each round of alignment was followed by principal component analysis and K-means classification, outputting 80 classes.

### Molecular modeling

Molecular modeling of the bacterial protein synthesis landscape was performed using the program Chimera [15]. For the 70S and 50S ribosome model, a 30-Å density map was calculated in the program spider using the x-ray crystal structure 1ML5 (pdb code) [16] as a template. A representative 30-Å density map for the *E.coli* RNA polymerase derived from 3LUO (pdb code) [4] was also calculated.

## Results and Discussion

### Recruitment of RNA-protein complexes onto Affinity Grids

**Figure 2:**
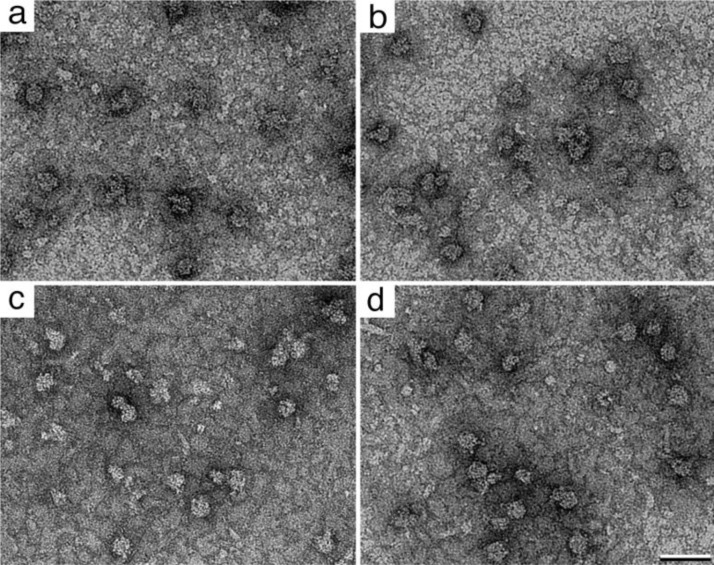
**Time course study for recruiting ribosomal complexes**. Images of negatively stained bacterial cell lysate samples incubated on 2% Ni-NTA Affinity Grids for **(a)** 2 minutes, **(b)** 5 minutes, **(c)** 10 minutes and **(d)** 20 minutes. Scale bar is 50 nm.

We used the bacterial protein synthesis machinery as a model system to test whether multiple RNA-binding components could be recruited onto Affinity Grid surfaces. We explored the feasibility of recruiting both translational and transcriptional complexes using a singular tag. We expressed his-tagged rpl3, the human homolog of a 46-kDa constituent of the 50S ribosomal subunit, in *E.coli* (BL21 strain) according to reference [11]. During culture growth, copies of the tagged subunit became incorporated into functional bacterial ribosomes. The bacterial cells were subsequently collected and lysed. His-tagged ribosomes were isolated onto Affinity Grids coated with a monolayer containing 2% Nickel-nitrilotriacetic acid (Ni-NTA) lipids and DLPC filler lipids. We envisioned that incubating cell lysate samples on the Affinity Grids for time periods longer than the standard 2-minute incubation time would increase our probability of recruiting a variety of machinery. Images of negatively stained specimens incubated for 2, 5, 10 and 20 minutes (Figure 2a - d) revealed complexes with dimensions representative of the 70S (∼35 nm) and 50S (∼ 30 nm) ribosomes.

Although there was a slight increase in the number of particles bound to the grid as the incubation time increased, there was also a slight increase in the level of non-specific bacterial contaminants bound to the grid. It was also difficult to visualize any particles in close proximity to the ribosomal complexes with characteristic features and dimensions of bacterial RNA polymerases (∼ 15 nm). There appeared to be a level of equilibrium established by the 10-minute incubation period and longer incubations did not show an appreciable increase in the number of complexes recruited to the grid. The level of complex recruitment is likely limited by the number of available Ni-NTA binding sites present in the lipid layer. Similarly, increasing the amount of Ni-NTA in the lipid layer beyond 2% increased the number of ribosomal complexes present on the layer, however, the number of particles became excessive and images were not suitable for processing. This result was consistent with previous observations [11].

**Figure 3:**
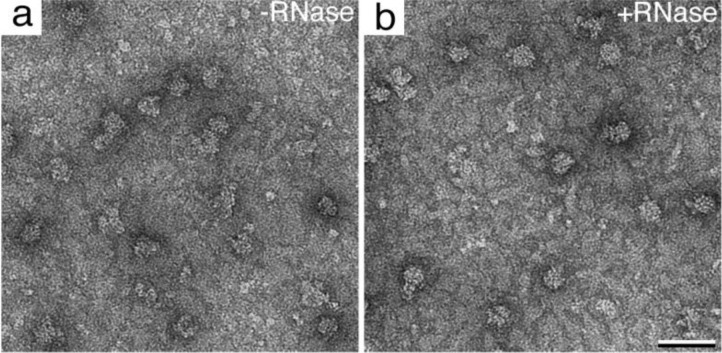
**Digestion of mRNA**. Bacterial lysates were incubated for 30 minutes in the absence **(a)** and presence **(b)** of RNase A. Scale bar is 50 nm. Samples incubated with RNase A show a more homogeneous population of translational complexes.

We postulated that RNA polymerases could be recruited by attaching to the nascent mRNA transcripts bound to recruited ribosomes. To test this idea, we incubated an aliquot of the bacterial lysate with 10 units of RNase A for 30 minutes at 4°C. Negatively stained samples of cell lysates incubated for 2 minutes upon the Affinity Grid with and without RNase A were prepared. Upon examining these specimens in the EM, we noted the complexes prepared in the presence of RNase A were more homogeneous (Figure 3b) than those prepared without RNase A (Figure 3a). This finding suggests that additional complexes may be present in the untreated sample. However, it was difficult to visually identify mRNA or polymerases in the raw images.

### Modifying the Affinity Grid surface

We attempted an alternative approach to stabilize mRNA and potentially recruit upstream transcriptional complexes to the grid surface. We theorized that by incorporating positively charged components into the lipid monolayer, we could better capture mRNA transcripts. The electrostatic complementarity between the negatively charged RNA backbone and the positively charged additive on the grid surface would likely be favorable (Figure 1). Stabilizing the nascent transcripts that mediate interactions between ribosomes and polymerases may serve to mediate pathway recruitment in our specimen preparation. However, a careful balance must be achieved to recruit His-tagged components specifically while stabilizing other attached complexes.

**Figure 4:**
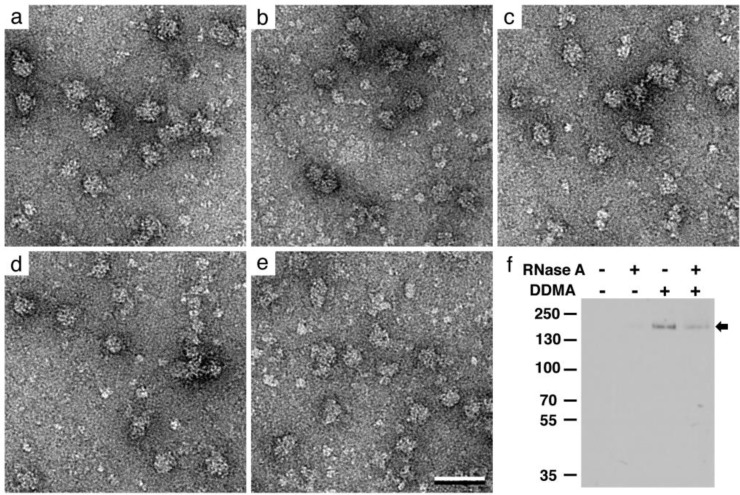
**The incorporation of DDMA onto Affinity Grid surfaces**. Images of negatively stained bacterial lysate samples added to 2% Ni-NTA Affinity Grids doped with **(a)** 0%, **(b)** 1%, **(c)** 2%, **(d)** 5% and **(e)** 10% DDMA. Scale bar is 50 nm. **(f)** Western blot detection of RNA Pol β (black arrow) with anti-RNA Polβ antibodies. The predicted molecular weight for RNA Pol β is ∼146 kDa.

DDMA is a lipophilic surfactant that carries a positive charge on its polar head group. It has been successfully used to promote 2D array formation of acidic cytoskeletal proteins based on charge complementarity [17–19]. In order to test whether the addition of DDMA into our lipid layer could stabilize RNA-tethered components, we prepared Affinity Grids coated with 2% Ni-NTA lipids along with 0, 1%, 2%, 5%, or 10% of DDMA. Aliquots of bacterial lysate were added to these modified grids, which were then negatively stained and imaged in the EM (Figure 4a - e). As the amount of DDMA was increased in the lipid layer, we observed an increase in the presence of what appears to be bacterial polymerases as well as other bacterial proteins. When 2 - 5% DDMA was added to the lipid layer, we observed an optimal number of assemblies having features consistent with RNA polymerases and adjacent to ribosomal complexes. Concentrations above 10% DDMA became saturated with particles (data not shown). We were, however, unable to visually confirm the presence of mRNA in our negatively stained specimens and chose to test for the presence of polymerases and mRNA biochemically.

To determine if RNA polymerases were being recruited to our Affinity Grids, we performed western blot analysis to probe for the presence of RNA Pol β, the 146-kDa core subunit of the polymerase (Figure 4f). Lysate samples were prepared in the presence and absence of RNase A and were applied to 20% Affinity Grids, with or without DDMA. Lysate samples were incubated on Affinity Grids for 2 minutes. Proteins recruited to the Affinity Grids were eluted with 200 mM imidazole and analyzed by western blot analysis. There was no detectable RNA polymerase captured on the grid in the absence DDMA (lane 1, Figure 4f). However, RNA polymerase could be detected in samples prepared on grids coated with monolayers containing both Ni-NTA and DDMA (lanes 3 and 4, Figure 4f). This supports the idea that including DDMA in the lipid layer coating on the Affinity Grid helps to facilitate the binding of RNA polymerases to the grid surface.

To test whether RNA polymerase recruitment was dependent upon the presence of mRNA, we examined eluates from lysate samples that were incubated with RNase A prior to applying to Affinity Grids. Eluates incubated with RNase A showed little to no RNA polymerases present on Ni-NTA containing grids lacking DDMA (lane 2, Figure 4f). However, there was an appreciable decrease in the amount of polymerase present in the lysate samples incubated with RNase A and prepared on grids containing both Ni-NTA and DDMA (lane 4, Figure 4f). This demonstrates that a majority of the RNA polymerases recruited to the Affinity Grids were bound to mRNA, which helps mediate attachment. We have previously demonstrated that no protein complexes were recruited to Affinity Grids lacking Ni-NTA [11]. We now provide strong evidence that RNA polymerases can be recruited to Ni-NTA containing Affinity Grids doped with positive charge via mRNA that associates with the grid surface and in the presence of ribosomes. This supports the idea that multiple complexes may be recruited onto Affinity Grids with modified surfaces.

### Visualizing multiple assemblies

**Figure 5:**
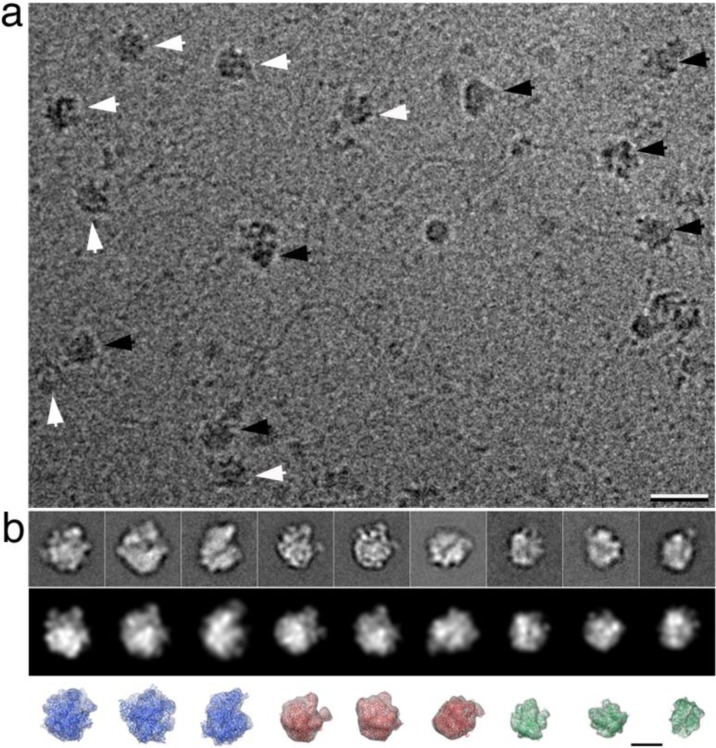
**Cryo-EM of RNA-binding complexes. (a)** Image of frozen-hydrated bacterial lysate prepared on modified Affinity Grids. A majority of ribosomal (black arrows) and polymerase complexes (white arrows) are bound to strands of mRNA. Scale bar is 25 nm. **(b)** Experimental averages for 70S ribosomes (top panel, 1 – 3), 50S ribosomes (top panel, 4 – 6) and RNA polymerase (top panel, 7 – 9) show a high correlation with theoretical projections (middle panel). Box size is 38 nm. Corresponding views of filtered density maps and crystals structures for the 70S ribosome (blue), 50S ribosome (red) and RNA polymerase (green) (bottom panel) show good agreement with the experimental projections. Scale bar is 15 nm. Contrast is inverted in **(b)**.

Previously, we visualized mRNA and genomic DNA in frozen-hydrated Affinity Grid specimens of human RNA polymerase II [20]. In order to determine in our present work if mRNA was physically connecting the recruited components, we prepared vitrified specimens and examined them using cryo-EM. We again titrated increasing amounts of DDMA into the lipid monolayer ranging from 0 – 50%. Since more Ni-NTA is needed to properly capture His-tagged components on the Affinity Grid, we chose to test a higher range of incorporating DDMA into our lipid layer (data not shown). The optimum level of complex recruitment was obtained using a lipid layer of 20% Ni-NTA lipid along with 10% DDMA and DLPC filler lipid (Figure 5a). Images of vitrified specimens reveal the presence of ribosomal complexes (Figure 5a, black arrows) attached to strands of mRNA that may preserve the connectivity to transcriptional complexes (Figure 5a, white arrows).

We recorded 200 images of vitrified specimens and selected 12,510 individual particles using the WEB interface of the SPIDER software package [14]. The selected particles were subjected to multi-reference alignment and classification routines using SPIDER. Representative averages (Figure 5b, top panel) were compared to projections filtered at 25-Å resolution for 70S and 50S ribosomes (derived from 1ML5, pdb code [16]) as well as RNA polymerase (derived from 3LUO, pdb code [4]) (Figure 5b, middle panel). A normalized cross-correlational routine was implemented to compare features of the experimental averages with those of the theoretical projections. Experimental averages showed on average a high cross-correlational coefficient of 0.8 (on a scale of 0 – 1.0) with respect to the theoretical projections. Additionally, a comparison of corresponding views for each filtered crystal structure with the experimental averages show good agreement (Figure 5b, bottom panel). Overall, this provides quantitative support that by using a modified lipid monolayer we are recruiting a heterogeneous population of potentially connected protein complexes containing both translational and transcriptional machinery.

### A new outlook on protein-nucleic acid interactions

**Figure 6:**
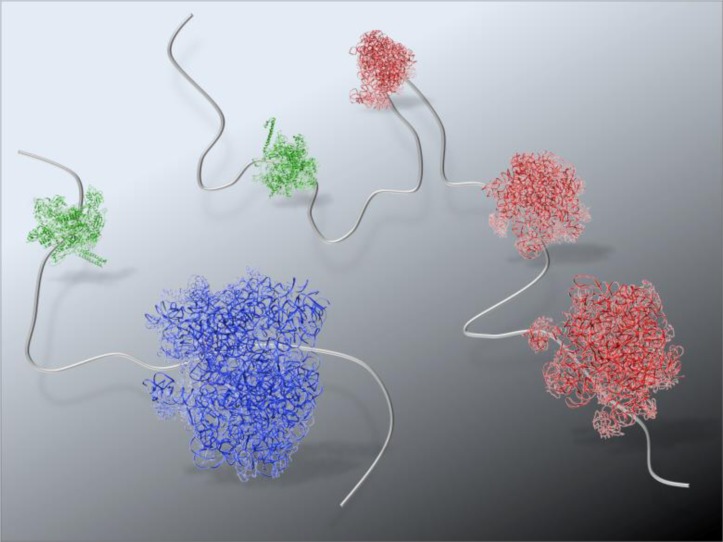
**Theoretical landscape of the bacterial protein synthesis machinery and mRNA**. Crystal structures for the *E.coli* 70s ribosome (blue, 1ML5) and 50S ribosome (red, 1ML5) attached to mRNA transcripts (gray strands) bound to RNA polymerases (green, 3LUO). This theoretical landscape, although speculative, is based on the image information in Fig. 5a (upper half of image).

We are currently using cryo-Electron Tomography to examine the intricate connections of biological pathways. We can meanwhile propose a theoretical model for protein synthesis based on our recent findings. The relationship between mRNA and ribosomal complexes in various states has been previously determined to sub-nanometer resolution (reviewed in reference [5]). Likewise the topology of *E.coli* RNA polymerase with respect to mRNA is also known [4]. We have, therefore, combined this information with our cryo-EM projection data (Figure 5a, upper half of image) to calculate an initial theoretical model for how transcription and translation may be coupled in bacteria (Figure 6). This molecular landscape, albeit speculative, gives rise to a new outlook for visualizing biological pathways. The inclusion of other key players that mediate these connections will be incorporated into a theoretical landscape as we acquire electron tomography data.

The notion of transcription-coupled translational events in prokaryotes is not a novel biological finding. The structural evidence for these connections originates from the elegant work of Miller and coworkers [21]. The authors used the classical rotary-shadowing technique to prepare EM specimens of intact bacterial genetic material. The potential molecular connections shown in our work are consistent with these classical findings. The shadowing preparation method involves applying biological samples onto EM grids, allowing the grids to air-dry then depositing a fine particulate of heavy metal to the grid. This technique is widely used to demonstrate connections between protein complexes and nucleic acids [22–25]. The major disadvantages with the shadowing method are the loss of fine molecular details and the flatness introduced to the specimen during the drying process.

To date, cryo preservation by plunging into liquid ethane remains the best method to prepare biological specimens for EM structural analysis [26]. Our work demonstrates the use of affinity capture techniques combined with cryo preparation preserves the molecular architecture of multi-component biological systems while maintaining them in a hydrated state. An additional benefit of our system is the localization of fragile biological assemblies into the holes of perforated EM carbon films. This successfully harbors protein machinery away from the disruptive surface tension forces at the air-water interface during vitrification [27].

The ability to simultaneously capture multiple players of cellular pathways opens a new avenue in structural biology. This would permit us to examine functional protein machinery and connections at unprecedented resolution. Furthermore, we could potentially devise a molecular blueprint to design therapeutic agents for RNA-dependent processes in pathogenic bacteria. Overall, the Affinity Capture platform may be used to visualize native cellular pathways within a single image. We anticipate this approach will improve our present understanding of how cellular processes coordinate biological systems.
